# Treatment of Visceral Leishmaniasis: Model-Based Analyses on the Spread of Antimony-Resistant *L. donovani* in Bihar, India

**DOI:** 10.1371/journal.pntd.0001973

**Published:** 2012-12-20

**Authors:** Anette Stauch, Hans-Peter Duerr, Jean-Claude Dujardin, Manu Vanaerschot, Shyam Sundar, Martin Eichner

**Affiliations:** 1 Department of Medical Biometry, University of Tuebingen, Tuebingen, Germany; 2 Laboratory of Microbiology, Parasitology and Hygiene, University of Antwerp, Antwerp, Belgium; 3 Department of Biomedical Sciences, Institute of Tropical Medicine Antwerp, Antwerp, Belgium; 4 Institute of Medical Sciences, Banaras Hindu University, Varanasi, India; Oswaldo Cruz Foundation, Brazil

## Abstract

**Background:**

Pentavalent antimonials have been the mainstay of antileishmanial therapy for decades, but increasing failure rates under antimonial treatment have challenged further use of these drugs in the Indian subcontinent. Experimental evidence has suggested that parasites which are resistant against antimonials have superior survival skills than sensitive ones even in the absence of antimonial treatment.

**Methods and Findings:**

We use simulation studies based on a mathematical *L. donovani* transmission model to identify parameters which can explain why treatment failure rates under antimonial treatment increased up to 65% in Bihar between 1980 and 1997. Model analyses suggest that resistance to treatment alone cannot explain the observed treatment failure rates. We explore two hypotheses referring to an increased fitness of antimony-resistant parasites: the additional fitness is (i) disease-related, by causing more clinical cases (higher pathogenicity) or more severe disease (higher virulence), or (ii) is transmission-related, by increasing the transmissibility from sand flies to humans or vice versa.

**Conclusions:**

Both hypotheses can potentially explain the Bihar observations. However, increased transmissibility as an explanation appears more plausible because it can occur in the background of asymptomatically transmitted infection whereas disease-related factors would most probably be observable. Irrespective of the cause of fitness, parasites with a higher fitness will finally replace sensitive parasites, even if antimonials are replaced by another drug.

## Author Summary

The protozoan flagellate *Leishmania donovani* causes the neglected, life-threatening disease visceral leishmaniasis. Parasites are transmitted from man to man by the bite of the sand fly *Phlebotomus argentipes*, the vector of the disease. Pentavalent antimonials have been the mainstay of antileishmanial therapy for decades but rapidly increasing failure rates up to 65% observed between 1980 and 1997 in the state of Bihar, India, have challenged further use of these drugs. Comparative *in vitro* and *in vivo* experiments indicate that antimony-resistant parasites have a higher fitness than antimony-sensitive ones even in the absence of antimonial treatment. Simulation studies based on a previously published mathematical *L. donovani* transmission model suggest that resistance to antimonial treatment alone cannot explain the Bihar observations but that resistance together with higher fitness offers the potential to explain the data. After an antimony-resistant parasite with higher fitness has emerged, it will finally replace the antimony-sensitive ones, even in complete absence of antimonial treatment.

## Introduction

Visceral leishmaniasis (VL), also known as Kala-azar (KA), causes each year about 200,000 to 400,000 cases with about 20,000 to 40,000 deaths worldwide. About 70% of the VL burden occurs in the Indian subcontinent, mostly in the state of Bihar [1]. The disease is caused by the infection with the protozoan flagellate *Leishmania donovani*. Most infections do not lead to clinical symptoms but progress asymptomatically [2]. In case of a symptomatic course of disease the infection is lethal in the absence of treatment.

Pentavalent antimonials were introduced in 1922 as therapeutic drug for leishmaniasis [3]. They remained the first-line treatment for about 70 years and had a treatment success rate of up to 95% [4]. Since the early 1980s, poor treatment responses have increasingly been reported from Bihar [5], [6], resulting in WHO's recommendations to increase treatment dosage and duration. Although this initially improved the results, the effects were only temporary [7]–[9]. The worst treatment outcome for pentavalent antimonials was reported from Bihar in 1997 with a treatment success rate of only 35% [10]. We refer to this rapidly increasing treatment failure rate (TFR) in Bihar in the following as ‘the Bihar data’. Increased treatment failure has also been reported from Nepalese districts neighbouring Bihar [11], [12]. In *in vitro* tests a reduced sensitivity to pentavalent antimonials could be demonstrated with mice-derived macrophages infected with *L. donovani* parasites obtained from non-responsive patients [13].

In 2005, the governments of India, Nepal, and Bangladesh agreed to participate in a regional ‘VL elimination program’ to reduce the annual incidence of VL from about 22 cases per 10,000 inhabitants to only one case per 10,000 inhabitants by 2015. This programme was based among others on the replacement of antimonials as first-line treatment by the oral drug miltefosine. So far, the treatment success rate of this drug does not seem to be affected by antimony-resistance.

The action mechanism of antimonials is still poorly understood [14], but they seem to have a dual mode of action. On the one hand, they perturbate the redox-balance of the parasites [15] and on the other hand, they impose extra oxidative and nitrosative stress upon the parasite through interaction with the host cell [16], [17]. Recent molecular studies showed that (i) antimony-resistant parasites emerged from *L. donovani* populations with different genetic background [18] and (ii) that molecular mechanisms of drug resistance may vary with that genetic background [19]. Antimony-resistant *L. donovani* parasites can inhibit the patient's immune response even more than antimony-sensitive *L. donovani* [20] and are hereby able to prevent the drug from inducing an effective antileishmanial response through the host cell [21]. These adaptations seem to protect them not only against antimony-induced stress, but also against natural stress such that resistant parasites may have a higher general fitness under drug-free conditions than sensitive ones [22], [23].

The aim of this study is to investigate the dynamics of the emergence and spread of antimony-resistant *L. donovani* parasites in Bihar. Our overall work hypothesis is that antimony-resistance alone cannot explain the Bihar data and that increased fitness (also in absence of the drug) is required to support the observed data. We use the word ‘fitness’ to describe the potential of the pathogen to promote its survival, its reproduction and its transmission [24], [25]. Within our model analyses, we assume that the increased fitness of antimony-resistant parasites is a stable condition despite the fact that the exact mechanisms behind this are not fully understood.

The first mathematical study of the dynamics of VL used a deterministic model to explain the observed inter-epidemic periods between 1875 and 1950 in Assam, India [26]. This model was extended to canine VL in Malta [27], [28] to assess the efficacy of various control methods [29]. This model and subsequently developed models have been applied to describe dynamics of canine VL in Brazil, in particular addressing the effects of interventions like culling dogs or using impregnated collars [30]–[32].

Based on data from Morocco, a mathematical model for cutaneous leishmaniasis was developed [33]. The model takes into account a latent period in humans and seasonal vector abundance. To study the dynamics of visceral leishmaniasis in the Sudan, a deterministic model was used to establish threshold conditions for elimination and to investigate the role of cross-immunity [34], [35]. Related to the data used in the present investigation the problem of under-reporting VL incidence in Bihar, India, has been investigated [36]. Recent modelling approaches addressed the effects of vaccination coverage and human migration on control of VL [37] and disease progression in mice [38] Using our previously published mathematical model for *L. donovani* transmission in the Indian subcontinent [39], we investigate which parameter constellations are capable to explain the observed dynamics of the TFR in humans treated with antimonials in Bihar. Following experimental findings, we explore five scenarios on higher fitness in addition to antimony-resistance. On the one hand, resistant parasites survive more likely within macrophages [40] and produce higher parasite burdens in the liver and the spleen of mice [23]. This may indicate that resistant parasites have a higher pathogenicity or virulence in human hosts. A higher capability to survive killing mechanisms of macrophages [40] could lead to more clinical cases, or to more severe disease. We will explore these possibilities as scenarios 1 ( = more clinical cases) and 2 ( = more severe disease) which are summarised as ‘disease-related fitness hypothesis’. On the other hand, it has been shown that resistant parasites have a higher metacyclogenic capacity during *in vitro* promastigote growth (which naturally occurs in the vector). The infectious metacyclic parasite is non-sensitive to complement lysis in the human host [41], which might increase the infection probability in humans. It might also increase the parasite density during early human infection or during the entire course which would consequently increase the infectiousness of asymptomatic human carriers or of all humans with resistant parasites. We will explore these possibilities as scenarios 3 ( = increased infectiousness in asymptomatic humans), 4 ( = increased infectiousness in all infected humans) and 5 ( = increased infectiousness in sand flies) which are summarised as ‘transmission-related fitness hypothesis’.

## Methods

**Figure 1:**
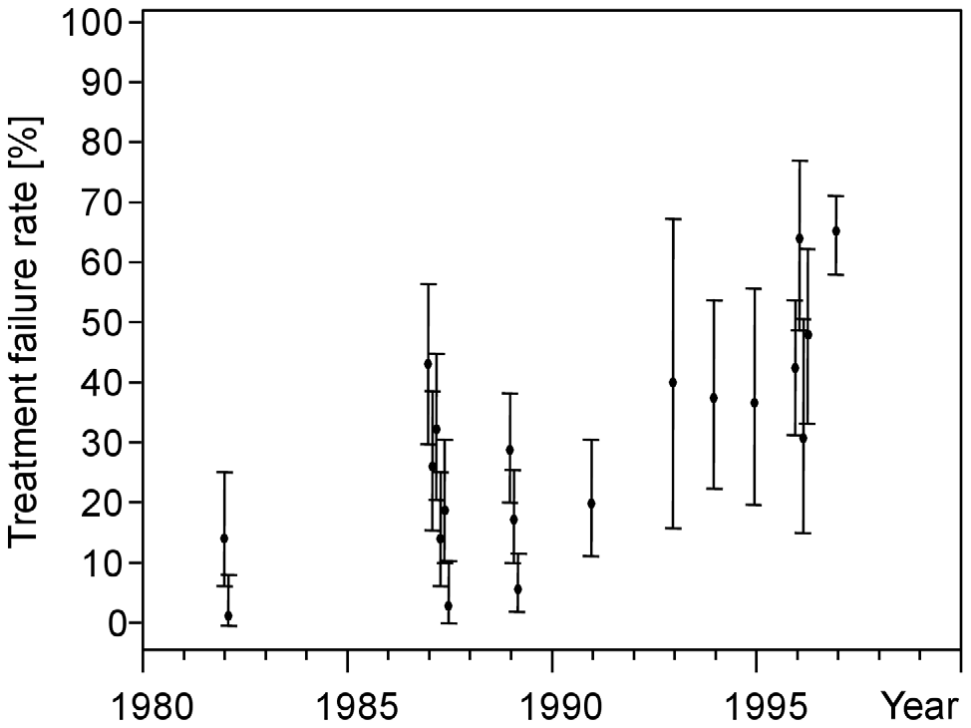
Data. Twenty treatment groups taken from 12 clinical studies involving antimonial treatment in Bihar, India, between 1980 and 1997 [42]. Points and bars show observed treatment failure rates with 95% confidence intervals.

Data on the TFR of antimonial treatment originate from a review of clinical trials in Bihar between 1980 and 2004. Although treatment dosage and duration differed between studies over the study period of two decades the data support the finding of a substantially declining efficacy of antimonial treatment over time [42]. Caused by the alarmingly fast decline of the treatment efficacy, antimonials were abandoned in Muzaffarpur, a district of Bihar, after June 1997 [10]. Thus, we restrict our analyses to studies on antimonial treatment which were performed until 1997 as provided by the mentioned review. In Figure 1, the observed TFR in the 20 treatment groups of the 12 remaining clinical studies are shown together with their 95% confidence intervals.

We extend an existing mathematical model [39] by additional infection states representing infections with parasites resistant to antimonial treatment (see File S1, Figure S1, Tables S1, S2, S3, S4, S5, S6, and S7). In the following, the expression ‘resistant parasite’ is used as a short form for ‘a parasite which is less susceptible to antimonial treatment and thus, increases the TFR’.

We vary parameters controlling the longitudinal evolution of the TFR and select all those parameter combinations for which the simulated TFR curves pass through the confidence intervals in Figure 1; if more than one data set is given for a time point, the simulation result has to pass through at least one of the corresponding confidence intervals. This analysis comprises parameters *T*, *r*, *f_H_*, *f_FS_*, *f_FA_*, *f_FH_*, *f_HF_* which are described as follows.

Simulations start in the endemic equilibrium with only sensitive parasites, using a population of 104 millions inhabitants (population size of Bihar). The mathematical model is considered to be in the endemic equilibrium when there is no change in the variables over a period of at least 100 years within an accuracy of five digits after the decimal (see Figure S1 and Tables S1 and S2). Parameter *T* is the year when the first resistant parasite emerges in a single patient.

Parameter *r* controls the TFR of patients infected with the resistant strain; it ranges between 0 and 1. For *r* = 0, the TFR is as high for carriers of the resistant strain (*TFR_res_*) as for carriers of the sensitive one (*TFR_sens_*; i.e. 5%), and for *r* = 1, treatment fails in all patients infected with the resistant strain. Thus, *TFR_res_* is given by *TFR_sens_*+*r* (1−*TFR_sens_*). For purposes of interpretation, parameter *r* will be reported throughout this manuscript in terms of *TFR_res_*. The observed TFR in the population is a weighted average of the TFR of patients infected with the sensitive strain and that of patients infected with the resistant one.

We consider five scenarios with modified fitness of resistant parasites:

1. Resistant parasites increase the probability that infected humans become sick. This increases the transmission probability of the parasites because of the long infectious duration and the high transmissibility of patients. The proportions 0.28% and 0.01% who develop KA or post-kala-azar dermal leishmaniasis (PKDL), respectively, when infected with sensitive parasites (see parameters *f_HS_* and *f_HL_* in Table S4) are multiplied with the pathogenicity factor *f_H_*: for *f_H_*>1, more patients with resistant parasites become sick, for *f_H_*<1, less become sick.
2. Resistant parasites increase the parasite load in patients due to a higher virulence. This increases the probability that sand flies become infected when feeding on patients. The probability of 10% that they become infected when feeding on a patient with sensitive parasites (see parameters *p_F3_* and *p_F4_* in Table S4) is multiplied with the virulence factor *f_FS_*: for *f_FS_*>1, more sand flies become infected when feeding on symptomatic hosts of resistant parasites, for *f_FS_*<1, less become infected.
3. Resistant parasites increase the probability that sand flies become infected when feeding on asymptomatically infected hosts. The probabilities 2.2% and 4.4% that they become infected when feeding on a host with sensitive parasites who is in the early or late infected stage, respectively, (see parameters *p_F1_* and *p_F2_* in Table S4) are multiplied with the transmissibility factor *f_FA_*: for *f_FA_*>1, more sand flies become infected when feeding on asymptomatic hosts of resistant parasites, for *f_FA_*<1, less become infected.
4. Resistant parasites increase the probability that sand flies become infected when feeding on symptomatic or asymptomatic hosts. The probabilities that they become infected when feeding on a host with sensitive parasites (parameters *p_F1_*, *p_F2_*, *p_F3_* and *p_F4_*) are multiplied with the transmissibility factor *f_FH_*: for *f_FH_*>1, more sand flies become infected when feeding on hosts with resistant parasites, for *f_FH_*<1, less become infected.
5. Resistant parasites increase the probability that humans become infected when being bitten by infected sand flies. The probability of 10% that a human becomes infected with the sensitive parasite (see parameters *p_H_* in Table S4) is multiplied with the transmissibility factor *f_HF_*: for *f_HF_*>1, more humans become infected with resistant parasites, for *f_HF_*<1, less become infected.

We numerically solve the differential equation model for 700,000 sets of parameter values and calculate how the TFR changes over time because of the spread of resistant parasites. Apart from using the standard parameter values given in Tables S3, S4, S5, and S6, we employ uniformly distributed random parameter values: parameters *T* (range 1922–1980) and *TFR_res_* (range 5–100%) are always varied; concomitantly, we pick one of the fitness parameters *f_H_* (range 0–10), *f_FS_* (range 0–10), *f_FA_* (range 0.7–1.3), *f_FH_* (range 0.7–1.3) or *f_HF_* (range 0.7–1.3) while the others are set to 1.

## Results

**Figure 2:**
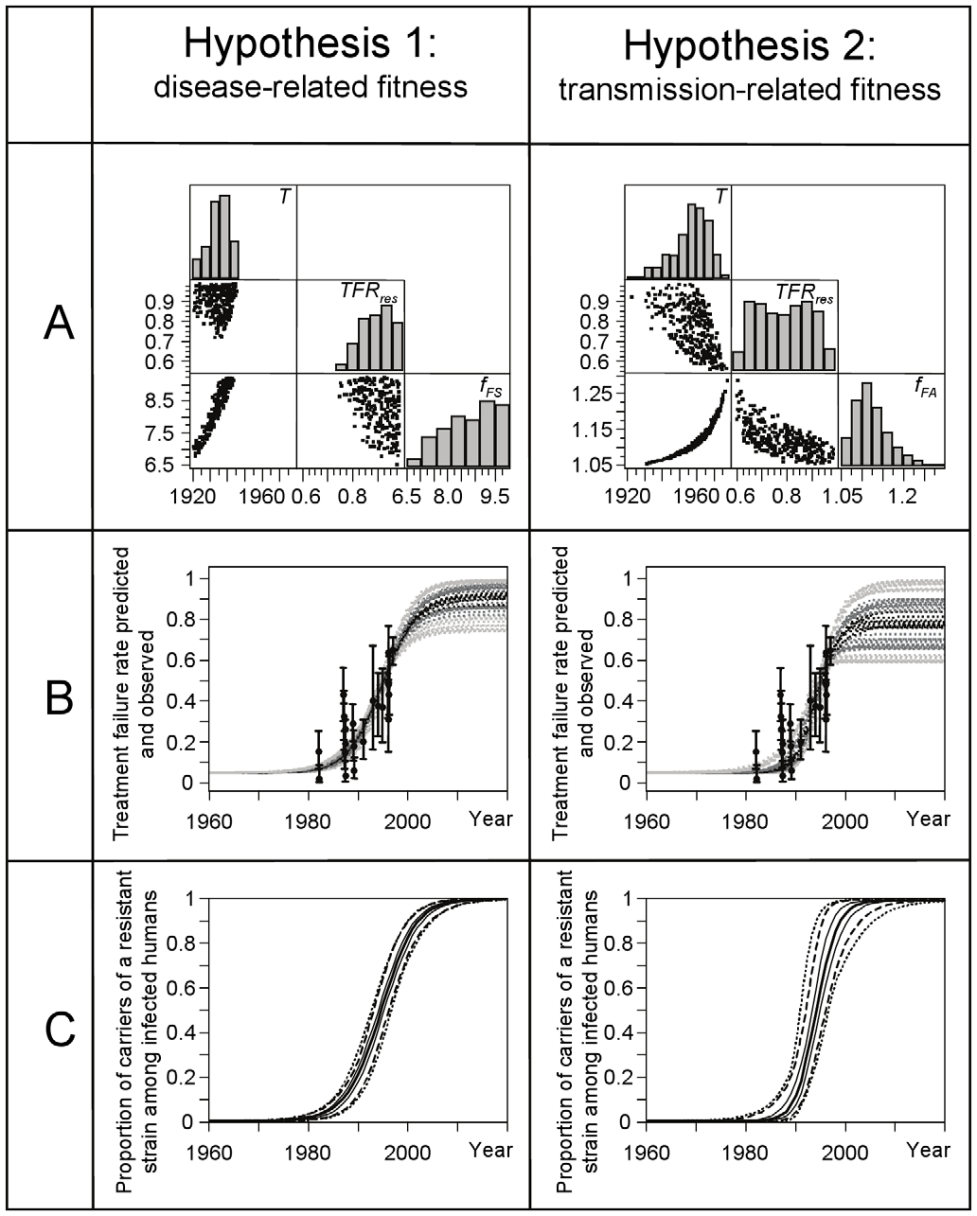
Analyses on the effects of parameter variations on the treatment failure rates observed in Bihar. Two hypotheses on increased fitness in resistant parasites are compared in three panels A, B, C. Panel A) the distributions of and correlations between *T* (the year when first resistance has emerged), *TFR_res_* (the treatment failure rate of patients infected with the resistant strain) and *f_FS_* (the disease-related fitness factor) or *f_FA_* (the transmission-related fitness factor). Panel B) the simulated overall TFR predicted by the model together with the data based observed TFR and their 95% confidence intervals. Panel C) the predicted proportions of resistant infections among all infections. In panel B and C, distributions of the hypothesis-specific predictions are represented by the minimum, by quantiles 2.5%, 25%, 50%, 75%, and 97.5%, and by the maximum. Model predictions are extrapolated until 2020 under the assumption of an unchanged antimonial treatment schedule.

Among 700,000 parameter combinations, the TFR curves of 1,605 combinations pass through the confidence intervals shown in Figure 1. These 1,605 parameter combinations are shown as scatter plots in Figure 2A. Quantiles of the simulated time-dependent TFR of these combinations are shown together with the field observations (Figure 1) and their 95% confidence intervals in Figure 2B. Figure 2C shows quantiles of the predicted proportions of resistant infections among all infections.

The TFR of cases infected with resistant parasites (*TFR_res_* Figure 2A) must be over 60% in order to explain the Bihar observations. Roughly 20 to 40 years after the first noticeable decline in treatment efficacy, sensitive parasites are nearly replaced by resistant ones (Figure 2C). As a consequence, the predicted treatment failure rate increases from 5% to *TFR_res_* (Figure 2B). An unchanged antimonial treatment schedule will always lead to strain replacement (Figure 2C). Without additionally increasing the fitness of resistant parasites, neither a *TFR_res_* of 60% nor even a *TFR_res_* of 100% can explain the observed TFR. We investigated two hypotheses for such an increased fitness:

### Hypothesis 1: additional fitness is disease-related

Resistant pathogens have a higher fitness because they lead to more clinical cases (scenario 1: pathogenicity factor *f_H_*>1) or to more severe disease (scenario 2: virulence factor *f_FS_*>1) and are, thus, more likely transmitted. As the results of these scenarios are almost undistinguishable, we show only the results of scenario 2 in Figure 2. To explain the Bihar data, one of the two disease-related fitness parameters *f_H_* or *f_FS_* must be larger than 6.5 (see *f_FS_* in Figure 2A). This means that resistant infections cause at least 6.5 times as many symptomatic cases compared to sensitive infections, or that clinical cases infected with resistant parasites infect 6.5 times as many sand flies. In the disease-related fitness scenarios, the TFR of patients infected with resistant parasites must be larger than 70%, and it must be assumed that the resistance emerged before 1950 (Figure 2A).

### Hypothesis 2: additional fitness is transmission-related

Resistant pathogens have a higher fitness because they have a higher transmissibility in asymptomatic carriers (scenario 3: *f_FA_*>1), in all human carriers (scenario 4: *f_FH_*>1), or in sand flies (scenario 5: *f_HF_*>1). As the results of these scenarios are almost undistinguishable, we show in Figure 2 only the results of scenario 3. To explain the Bihar data, one of the three transmissibility factors *f_FA_*, *f_FH_* or *f_HF_* must be larger than 1.05 (see *f_FA_* in Figure 2A). This means that the transmissibility in asymptomatic human carriers, in all human carriers, or in sand flies must increase by at least 5%. This minor increase strongly impacts the transmission dynamics because of the large proportion of asymptomatic infections (see Discussion). In the transmission-related fitness scenarios, the TFR of patients infected with resistant parasites must be larger than 60%, while the time point of emergence of resistance can hardly be determined (emergence between 1922 and 1979) (Figure 2A).

In summary, the explanation of the Bihar data requires the assumptions that the TFR of patients infected with resistant parasites is high and that resistant parasites have an increased fitness. Both hypotheses on additional fitness offer the potential to explain these data.

## Discussion

Using a mathematical model for *L. donovani* transmission, we have identified parameter sets which can explain the antimonial treatment failure rates observed in Bihar between 1980 and 1997. The simulations suggest that antimony-resistance alone cannot explain the observed rapid increase in TFR if *L. donovani* transmission is largely driven by asymptomatic carriers [39] which are not exposed to a selection pressure originating from antimonial treatment. Even if all patients infected with antimony-resistant strains would be treatment failures (*TFR_res_* = 100%), the observed TFR in Bihar cannot be explained. Noteworthy, parameter *TFR_res_* is likely to be even lower in reality, close to 60%, as shown in Nepal where antimony-resistant parasites were identified in most cases of treatment failure, but are also found in half the patients who showed definite cure [43].

Two hypotheses on increased fitness can explain the Bihar observations.

Hypothesis 1: a disease-related fitness with *f_H_* or *f_FS_* ranging from 6.5 to 10 means that resistant parasites produce 6.5 to 10 times more symptomatic cases, or that symptomatic carriers of resistant parasites are 6.5 to 10 times more infectious than symptomatic carriers of sensitive parasites. For reasons described below we regard this as the less plausible explanation.

Hypothesis 2: a transmission-related fitness with *f_FA_*, *f_FH_* or *f_HF_* ranging from 1.05 to 1.30 means that the transmissibility by the vector or by the host (i.e. asymptomatically infected or all infected humans) increases by 5% to 30%. For reasons described below we regard this as the more plausible explanation.

To demonstrate the effects of the increase in disease-related fitness parameters (Hypothesis 1 in Figure 2), or transmission-related fitness parameters (Hypothesis 2 in Figure 2), we calculated equilibrium solutions of the model, obtained by those parameter sets which can potentially explain the Bihar observations. From these parameter sets, we choose five representatives (using the median of each accepted fitness parameter while keeping *TFR_res_* = 100%) and compare the obtained equilibrium solutions with a scenario without any resistance. Higher pathogenicity of antimony-resistant parasites (*f_H_* = 8.7) would lead to an approximately 15-fold prevalence of KA and an approximately 8-fold prevalence of PKDL. Such an excessive increase of symptomatic cases is likely to be clinically observable and to our knowledge there is no clinical evidence for such an increase. For the other four parameter sets (*f_FS_* = 8.9, *f_FA_* = 1.12, *f_FH_* = 1.12 or *f_HF_* = 1.12), the KA prevalence would be only approximately doubled.

In case of higher virulence, the strongly increased infectiousness of symptomatic cases infected with resistant parasites might be confirmed by xenodiagnosis or by quantitative PCR of blood or skin tissue of the patients. *In vivo* data in mice infected with antimony-resistant parasites show a 3- to 8-fold higher parasite burden in liver and spleen [23]. This finding would support a disease-related fitness hypothesis if a higher parasite burden implied higher infectiousness and if antimony-resistant parasites had comparable properties in humans and mice. Up to now there is no clinical evidence for more severe disease in patients. However, higher parasite loads in patients may remain clinically undetectable because higher parasite loads do not necessarily lead to more severe symptoms. In case of the three transmission-related fitness scenarios, an increased transmissibility of 5% to 30% can definitely occur in the background of asymptomatic transmission without being recognized and thus may have a higher explanatory potential in this investigation.

We investigated disease- and transmission-related fitness parameters separately to quantify the effect of each parameter although combinations of several fitness parameters seem rather realistic as their biological origins are closely inter-linked.

Simulations of both hypotheses suggest that sensitive parasites are replaced almost completely by resistant ones already 20 to 40 years after the first noticeable decline in treatment efficacy (Figure 2C). To our knowledge there are no studies available in which random samples of strains were tested for their susceptibility to antimonials. Extrapolations from two studies in India and Nepal indicate a prevalence of antimony-resistant strains of around 65% at the beginning of the century [13], [43] while the analysis of 19 Indian strains collected in 2009 and 2010 in Bihar showed that 15 of them (thus 79%) were antimony-resistant [44]. To our knowledge the only available population-based non-response estimate in humans is from Muzaffarpur district, Bihar, 2008. Almost 10 years after antimonial treatment has been stopped still 67 of 131 retrospectively investigated VL patients report to have been treated with antimonials whereby treatment failed in 27 patients of them (40%, with 95% CI from 28 to 53%) [45]. From these data we may validate the abovementioned hypothesis by the following simple calculation. If we assume that about 79% [44] of the parasites are antimony-resistant and that treatment failed in about 60% of patients infected with resistant parasites [43] we would expect a TFR of about 47% which would lie within the 95% confidence limits of the above mentioned observed TFR of 40% [45].

Issues on selection pressure in the context of antimony resistance are complex. Poor treatment compliance is suspected to have caused the development of resistance in Bihar [46]. Accordingly, under-dosage of antimonials, which is not lethal to the parasite, may have created conditions under which the development of resistance provides selection benefits. Additional factors might have contributed to antimony resistance, like arsenic contamination of the groundwater [47]. Groundwater as drinking water has become available via tubewells in the 1970s, just before antimonial treatment failures started to increase. The geographic distribution of contaminated tubewells correlates with high rates of antimonial treatment failure. As metalloids, antimony and arsenic share biochemical features. Chronic exposure of a large proportion of the population to arsenic with antileishmanial properties might have contributed to the establishment of a resistant strain. An additional selection pressure might originate from the human host itself due to the nature of antimonial drugs which impose extra oxidative and nitrosative stress upon the parasite through interaction with the host cell [16], [17]. We currently cannot exclude that antimony resistance emerged on a background of parasites already fitter to their host. This is actually supported by the observation of abundant antimony-resistant *L. braziliensis* strains in Peru, in a context of zoonotic leishmaniasis where drug pressure is quasi null as most of the parasite bio-mass is in the wild animal reservoir [48].

An emergence of resistant parasites before 1960 seems rather unlikely: VL had almost been eliminated at that time as a consequence of the National Malaria Eradication program [49]. Although results of our analyses suggest that in case of a disease-related fitness factor, emergence must have occurred before 1950 (Figure 2A), this result will not be discussed in detail because it highly correlates with assumptions into multiple emergences of resistant parasites. In case of multiple emergences of resistance [18] and also in case of a contribution of arsenic contaminated groundwater on the spread of resistant parasites [47], our predictions must be understood as an upper limit for parameter *T*, the year when the first resistant parasite emerged in a patient.

Further data and model related limitations of these modelling analyses are pointed out in the following. To investigate the spread of antimony-resistant parasites we used a previously published mathematical model [39]. Model parameters were chosen from the literature or estimated by fitting the model to data from the KalaNet project, a community intervention trial in India and Nepal. Uncertainties originating from a high-dimensional parameter space and resulting correlations between parameters have been explored in that paper by means of sensitivity analyses

The main data related uncertainty originates from the assumption that cellular immunity can be represented by Leishmanin skin test (LST) measurements. Under the assumption of a life-long cellular immunity the model showed that the prevalence of LST-positive individuals in the population would be higher than 50% as had been observed (for further detail see [39]). Therefore, loss of LST-positivity had to be assumed, with re-infection occurring in intervals of about two years. In case that LST data do not adequately represent a status of protective immunity and that cellular immunity lasts longer than assumed resistant parasites would spread slower than suggested by this investigation.

Model related uncertainties originate from assumptions underlying the deterministic modelling approach as, for instance, homogeneous mixing within and between human and fly populations, an infinitely large population size, age structure of the human population, heterogeneities in living conditions, or seasonal transmission patterns. Such factors can influence short term predictions and would demand a stochastic modelling approach. As, however, this investigation addresses development of resistance over several decades we believe that stochastic influences are of minor relevance and that the deterministic model is adequate to describe a trend over decades.At least partially, antimonial treatment has been replaced in Bihar around the year 2000 by drugs like amphotericin B, miltefosin and paromomycin, which are assumed to be not affected by antimonial-related resistance. This leads to the question whether this will stop or even reverse the process of strain replacement, a question which might be of relevance if a return to antimonials in therapeutic schemes, including combination schemes, would be considered in the future. Thus, in addition to the model predictions in Figure 2C, which are produced under the assumption of unchanged treatment, we investigated scenarios in which antimonials are replaced by the above mentioned drugs around the year 2000. The predicted curves are visually indistinguishable from those shown in Figure 2C. Even if we assume that antimony-resistant parasites are less fit than sensitive ones in the presence of such a new drug the predicted curves are visually indistinguishable. In general, once a strain with higher fitness has emerged, it overgrows the ‘old’ one [50]. These findings are supported by *in vivo* data showing that antimony-resistant parasites overgrow antimony-sensitive parasites just few weeks after mice were co-infected with both phenotypes (PhD thesis MV, University of Antwerp).

## Conclusions

Our analyses suggest that antimony-resistance alone cannot explain why the TFR observed in Bihar increased up to 65% between 1980 and 1997. Most infections do not lead to symptomatic disease and thus, only a minority of parasites is exposed to antimonial treatment. This minor proportion of parasites cannot increase the TFR as quickly as it has been observed in Bihar.

Following recent experimental findings on increased fitness of resistant parasites, we examined two hypotheses for an additional fitness benefit: disease-related or transmission-related fitness increase. At the current stage of knowledge, we cannot favour one fitness hypothesis over the other; both offer the potential to explain the data. Disease-related fitness, however, requires increasing the proportion of clinical cases among all infections or the infectiousness of symptomatic cases by at least 550%, which most probably would have been observable.

Transmission-related fitness, on the other hand, requires increasing the infectiousness in sand flies, in asymptomatically infected humans, or in all infected humans by at least 5%. Such a minor increase can occur in the background of asymptomatic transmission without being apparently recognized under field conditions. After a parasite with higher fitness independent of a treatment-based selection pressure has emerged, it will finally replace the sensitive one, even in complete absence of antimonial treatment.

This modelling study suggests that entomological studies are urgently required to gain better data on sand flies abundance, biting rates and infectiousness. Furthermore, research on the fitness of the parasites should also be conducted in the context of the natural vector *Phlebotomus argentipes*, and last but not least, more research should be done on asymptomatic carriers and the type of parasites they carry, in order to weigh their role in transmission.

## Supporting Information

File S1 **Supplemental text.** A detailed description of the extended model is provided.
(DOC)

Figure S1 **Model for transmission of antimony-sensitive and antimony-resistant** ***L. donovani*** **parasites.** In addition to the previously published model [39], the compartments of humans and sand flies infected with antimony-resistant parasites are shown as a second layer.
(TIF)

Table S1 **Model variables—sand flies.**
(DOC)

Table S2 **Model variables—humans.**
(DOC)

Table S3 **Model parameters—sand flies** [**51]**–**[53]** **.**
(DOC)

Table S4 **Model parameters—humans** [**54]**–**[56]** **.**
(DOC)

Table S5 **Model parameters—treatment** [**57]**–**[60]** **.**
(DOC)

Table S6 **Model parameters—immuno-compromised humans** [**61**] **.**
(DOC)

Table S7 **Model parameters—resistance and fitness.**
(DOC)
