# Supplementary material for: Treatment of Visceral Leishmaniasis: Model-Based Analyses on the Spread of Antimony-Resistant L. donovani in Bihar, India
Source: PLoS Negl Trop Dis. 2012 Dec 20;6(12):e1973. doi: 10.1371/journal.pntd.0001973 (PMC3527335; doi:10.1371/journal.pntd.0001973)
Supplement: File S1 — Supplemental text. A detailed description of the extended model is provided. (DOC) [file pntd.0001973.s001.doc]

# File S1 - Supplemental text

## Description of the model

Transmission dynamics of *L. donovani* in the Indian subcontinent are modelled by a system of ordinary differential equations (see Figure S1and equations in the following section). Tables S1 to S7 explain the variables and parameters. In addition to our previously published *L. donovani* model, compartments of humans and sand flies infected with antimony-resistant parasites are implemented as a second layer of all parasite carrying compartments. All variables and parameters of hosts infected with resistant parasites have an additional index *r*. Parameters were estimated as described in detail in with following modifications: in this analysis we assume (i) a population of 104 millions inhabitants according to the population size of Bihar (ii) a probability of 0.1 that a susceptible human becomes infected after the blood meal of an infected sand fly and (iii) a probability of 0.1 that a sand fly becomes infected when feeding on KA or PKDL patients.

## Equations

### Humans

whereby $\mu_{T1}=f_{T}\cdot\tau_{1}$ and $\mu_{T2}=f_{T}\cdot\tau_{2}$

### Immuno-compromised humans

whereby $\mu_{T1}=f_{T}\cdot\tau_{1}$ and $\mu_{T2}=f_{T}\cdot\tau_{2}$ and $\begin{matrix} \alpha_{H}=\mu_{H}N_{H} \\ +\mu_{K}\left(I_{HS}+I_{HT1}+I_{HT2}+I_{VS}+I_{VT1}+I_{VT2}+I_{HSr}+I_{HT1r}+I_{HT2r}+I_{VSr}+I_{VT1r}+I_{VT2r}\right) \\ +\mu_{V}\left(S_{V}+I_{VP}+I_{VD}+R_{VD}+R_{VC}+I_{VS}+I_{VT1}+I_{VT2}+R_{VT}+R_{VL}+I_{VL}+I_{V\Pr}+I_{VDr}+I_{VSr}+I_{VT1r}+I_{VT2r}+R_{VLr}+I_{VLr}\right) \\ +\mu_{T1}\left(I_{HT1}+I_{VT1}+I_{HT1r}+I_{VT1r}\right) \\ +\mu_{T2}\left(I_{HT2}+I_{VT2}+I_{HT2r}+I_{VT2r}\right) \end{matrix}$

### Sand flies

$$\begin{matrix} \frac{dS_{F}}{dt}=\alpha_{F}-\left(\lambda_{F}+\lambda_{Fr}+\mu_{F}\right)S_{F} \\ \frac{dE_{F}}{dt}=\lambda_{F}S_{F}-\left(\sigma_{F}+\mu_{F}\right)E_{F} \\ \frac{dE_{Fr}}{dt}=\lambda_{Fr}S_{F}-\left(\sigma_{F}+\mu_{F}\right)E_{Fr} \\ \frac{dI_{F}}{dt}=\sigma_{F}E_{F}-\mu_{F}I_{F} \\ \frac{dI_{Fr}}{dt}=\sigma_{F}E_{Fr}-\mu_{F}I_{Fr} \end{matrix}$$

whereby $\alpha_{F}=\mu_{F}N_{F}$

### Infection rate λ

$$\begin{matrix} \lambda_{H}=\frac{\beta\cdot p_{H}\cdot I_{F}}{N_{H}} \\ \lambda_{Hr}=\frac{\beta\cdot p_{Hr}\cdot I_{Fr}}{N_{H}} \\ \lambda_{F}=\frac{\beta\left(p_{F1}\left(I_{HP}+I_{VP}\right)+p_{F2}\left(I_{HD}+I_{VD}\right)+p_{F3}\left(I_{HS}+I_{HT1}+I_{HT2}+I_{VS}+I_{VT1}+I_{VT2}\right)+p_{F4}\left(I_{HL}+I_{VL}\right)\right)}{N_{H}} \\ \lambda_{Fr}=\frac{\beta\cdot f_{FA}\left(p_{F1}\left(I_{H\Pr}+I_{V\Pr}\right)+p_{F2}\left(I_{HDr}+I_{VDr}\right)\right)+f_{FS}\left(p_{F3}\left(I_{HSr}+I_{HT1r}+I_{HT2r}+I_{VSr}+I_{VT1r}+I_{VT2r}\right)+p_{F4}\left(I_{HLr}I_{VLr}\right)\right)}{N_{H}} \end{matrix}$$

In case of scenario 4 in which resistant parasites can increase the probability that a sand fly becomes infected when feeding on symptomatic or asymptomatic hosts, fitness parameters *f_FA_* and *f_FS_* are replaced by fitness parameter *f_FH_*:
$\lambda_{Fr}=\beta\cdot f_{FH}\left(p_{F1}\left(I_{H\Pr}+I_{V\Pr}\right)+p_{F2}\left(I_{HDr}+I_{VDr}\right)+p_{F3}\left(I_{HSr}+I_{HT1r}+I_{HT2r}+I_{VSr}+I_{VT1r}+I_{VT2r}\right)+p_{F4}\left(I_{HLr}I_{VLr}\right)\right)$
