# Supplementary figures and images for: Treatment of Visceral Leishmaniasis: Model-Based Analyses on the Spread of Antimony-Resistant L. donovani in Bihar, India
Source: PLoS Negl Trop Dis. 2012 Dec 20;6(12):e1973. doi: 10.1371/journal.pntd.0001973 (PMC3527335; doi:10.1371/journal.pntd.0001973)

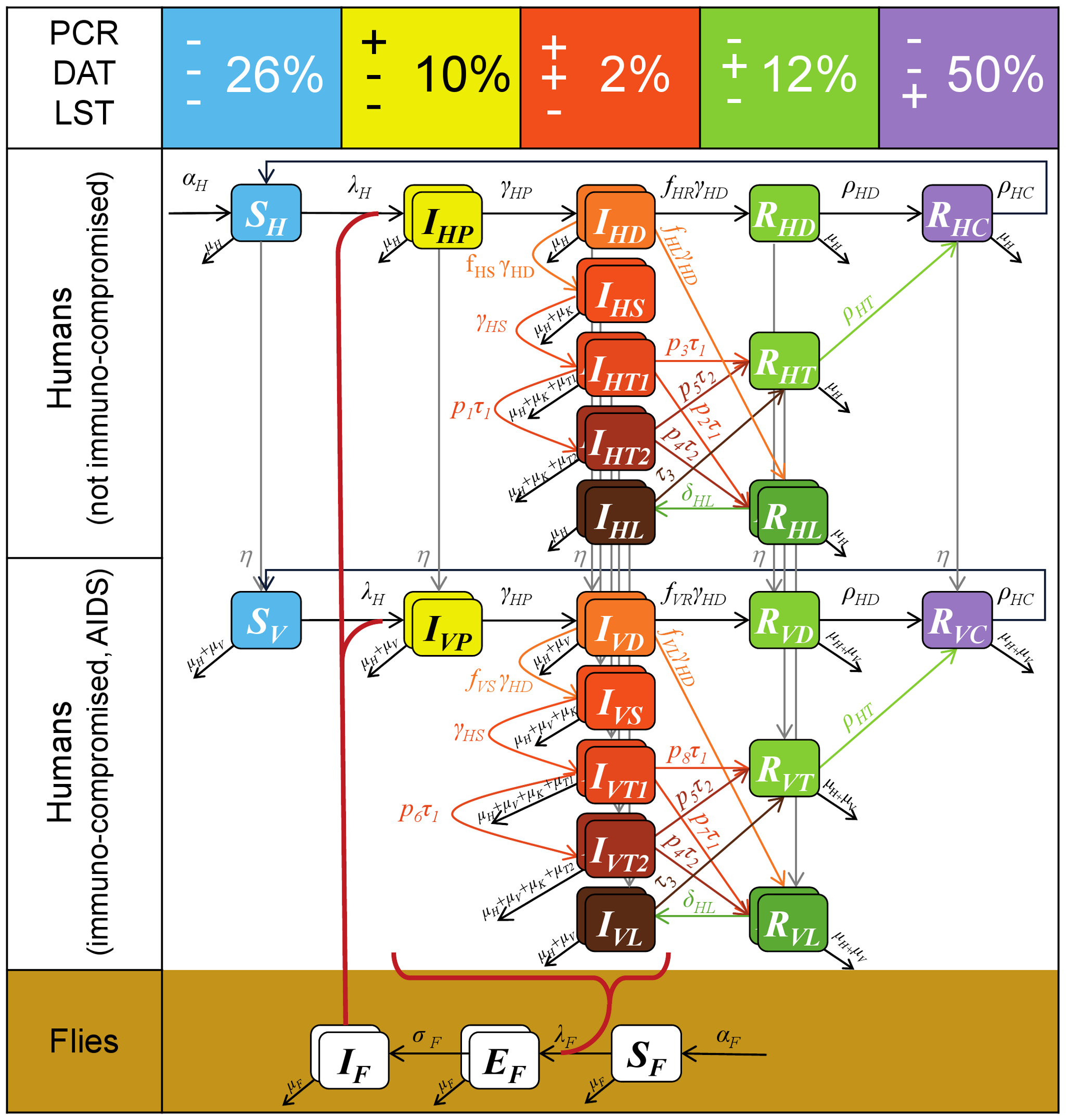

Supplement: Figure S1 — Model for transmission of antimony-sensitive and antimony-resistant L. donovani parasites. In addition to the previously published model [39], the compartments of humans and sand flies infected with antimony-resistant parasites are shown as a second layer. (TIF) [file pntd.0001973.s002.tif]
