# Supplementary material for: Treatment of Visceral Leishmaniasis: Model-Based Analyses on the Spread of Antimony-Resistant L. donovani in Bihar, India
Source: PLoS Negl Trop Dis. 2012 Dec 20;6(12):e1973. doi: 10.1371/journal.pntd.0001973 (PMC3527335; doi:10.1371/journal.pntd.0001973)
Supplement: Table S1 — Model variables—sand flies. (DOC) [file pntd.0001973.s003.doc]

## Table S1 – Model variables – sand flies.

| *S_F_* | Number of sand flies in the susceptible stage |
| --- | --- |
| *E_F_*, *E_Fr_* | Number of sand flies in the latent stage |
| *I_F_*, *I_Fr_* | Number of sand flies in the infectious stage |

Sand flies infected with antimony-resistant parasites are indicated by an additional index *r*.
