# Supplementary material for: Treatment of Visceral Leishmaniasis: Model-Based Analyses on the Spread of Antimony-Resistant L. donovani in Bihar, India
Source: PLoS Negl Trop Dis. 2012 Dec 20;6(12):e1973. doi: 10.1371/journal.pntd.0001973 (PMC3527335; doi:10.1371/journal.pntd.0001973)
Supplement: Table S2 — Model variables—humans. (DOC) [file pntd.0001973.s004.doc]

## Table S2 – Model variables – humans.

| *S_H_*, *S_V_* | Number of humans in the susceptible stage |
| --- | --- |
| *I_HP_*, *I_VP_*, *I_HPr_*, *I_VPr_* | Number of humans in the early asymptomatic, infectious stage |
| *I_HD_*, *I_VD_*, *I_HDr_*, *I_VDr_* | Number of humans in the late asymptomatic, infectious stage |
| *R_HD_*, *R_VD_* | Number of humans in the early recovered stage |
| *R_HC_*, *R_VC_* | Number of humans in the late recovered stage |
| *I_HS_*, *I_VS_*, *I_HSr_*, *I_VSr_* | Number of humans with symptomatic KA, eligible for treatment |
| *I_HT1_*, *I_VT1_*, *I_HT1r_*, *I_VT1r_* | Number of humans under first-line KA treatment |
| *I_HT2_*, *I_VT2_*, *I_HT2r_*, *I_VT2r_* | Number of humans under second-line KA treatment |
| *R_HT_*, *R_VT_* | Number of humans recovered after treatment |
| *R_HL_*, *R_VL_*, *R_HLr_*, *R_VLr_* | Number of humans putatively recovered under KA treatment but will develop PKDL |
| *I_HL_*, *I_VL_*, *I_HLr_*, *I_VLr_* | Number of humans with PKDL |

For immuno-compromised humans, index *H* is replaced by index *V*. Humans infected with antimony-resistant parasites are indicated with an additional index *r*.
