# Supplementary material for: Treatment of Visceral Leishmaniasis: Model-Based Analyses on the Spread of Antimony-Resistant L. donovani in Bihar, India
Source: PLoS Negl Trop Dis. 2012 Dec 20;6(12):e1973. doi: 10.1371/journal.pntd.0001973 (PMC3527335; doi:10.1371/journal.pntd.0001973)
Supplement: Table S3 — Model parameters—sand flies [51] – [53] . (DOC) [file pntd.0001973.s005.doc]

## Table S3 – Model parameters – sand flies.

|  | Description | Reference |
| --- | --- | --- |
| *N_F_* | Number of vectors: *N_F_* = 7638 million | Estimated |
| *μ_F_* | Mortality rate of sand flies, derived from life expectancy of sand flies: 1/*μ_F_* = 14 days |  |
| *α_F_* | Birth rate of sand flies: *α_F_* = *μ_F_* *N_F_* | Assumed |
| *σ_F_* | Rate determining the sojourn time of sand flies in stage *E_F_*, derived from 1/(*σ_F_* + *μ_F_*) = 5 days |  |
| *β* | Rate determining the feeding cycle duration, derived from 1/(*β* + *μ_F_*) = 4 days |  |
