# Supplementary material for: Treatment of Visceral Leishmaniasis: Model-Based Analyses on the Spread of Antimony-Resistant L. donovani in Bihar, India
Source: PLoS Negl Trop Dis. 2012 Dec 20;6(12):e1973. doi: 10.1371/journal.pntd.0001973 (PMC3527335; doi:10.1371/journal.pntd.0001973)
Supplement: Table S5 — Model parameters—treatment [57] – [60] . (DOC) [file pntd.0001973.s007.doc]

## Table S5 – Model parameters – treatment.

|  | Description | Reference |
| --- | --- | --- |
| *τ_1_* | Rate determining the sojourn time under first-line KA treatment, derived from 1/(*τ_1_*+*μ_H_*+*μ_K_*) = 30 days |  |
| *τ_2_* | Rate determining the sojourn time under second-line KA treatment, derived from 1/(*τ_2_*+*μ_H_*+*μ_K_*) = 30 days |  |
| *τ_3_* | Rate determining the sojourn time under PKDL treatment, derived from 1/(*τ_3_*+*μ_H_*) = 180 days |  |
| *μ_T1_* | Excess mortality rate caused by first-line KA treatment, derived from *μ_T1_* = *f_T_* *τ_1_*, assuming that *f_T_* = 5% die because of treatment | Personal communication, MB, |
| *μ_T2_* | Excess mortality rate caused by second-line KA treatment, derived from *μ_T2_* = *f_T_* *τ_2_*, assuming that *f_T_* = 5% die because of treatment | Personal communication, MB, |
| *p_1_* | Proportion of KA patients not responding to KA first-line treatment, derived from *p_1_* = (1-*f_T_*) *f_1_*, assuming that *f_1_* = 5% of KA patients who are not killed by the treatment, do not respond to KA first-line treatment |  |
| *p_2_* | Proportion of KA patients who appear to recover under KA first-line treatment but will develop PKDL later, derived from  *p_2_* = (1-*f_T_*)(1-*f_1_*) *f_2_*, assuming that a fraction *f_2_* = 3% of KA patients who are neither killed by the treatment nor experienced obvious treatment failure appear to recover under KA treatment but will develop PKDL | Personal communication, MB, |
| *p_3_* | Proportion of KA patients recovering during KA first-line treatment, derived from *p_3_* = (1-*f_T_*)(1-*f_1_*)(1-*f_2_*) |  |
| *p_4_* | Proportion of KA patients who appear to recover under KA second-line treatment but will develop PKDL later, derived from  *p_4_* = (1-*f_T_*) *f_2_*, assuming that a fraction *f_2_* = 3% of KA patients who are not killed by the treatment appear to recover under KA treatment but will develop PKDL |  |
| *p_5_* | Proportion of KA patients recovering during KA second-line treatment, derived from *p_5_* = (1-*f_T_*)(1-*f_2_*) |  |
| *δ_HL_* | Rate determining the sojourn time in stage *R_HL_*, derived from 1/(*δ_HL_*+*μ_H_*) = 21 months |  |
