# Supplementary material for: Treatment of Visceral Leishmaniasis: Model-Based Analyses on the Spread of Antimony-Resistant L. donovani in Bihar, India
Source: PLoS Negl Trop Dis. 2012 Dec 20;6(12):e1973. doi: 10.1371/journal.pntd.0001973 (PMC3527335; doi:10.1371/journal.pntd.0001973)
Supplement: Table S6 — Model parameters—immuno-compromised humans [61] . (DOC) [file pntd.0001973.s008.doc]

## Table S6 – Model parameters – immuno-compromised humans.

|  | Description | Reference |
| --- | --- | --- |
| *μ_V_* | Excess mortality rate in immuno-compromised patients, assuming *μ_V_* = 0.2/year | Assumed |
| *η* | Rate of developing AIDS or other immuno-compromising disease *η*= 1.9 x 10^-6^ day | Estimated |
| *f_VS_* | Fraction of individuals in stage *I_VD_* who develop symptomatic KA: *f_VS_* = 0.034 | Estimated |
| *f_VL_* | Fraction of individuals in stage *I_VD_* who go directly to *R_VL_* and will later develop PKDL: *f_VL_ = f_HL_* | Assumed |
| *f_VR_* | Fraction of individuals in stage *I_VD_* who recover without showing a symptomatic course of infection (→*R_VD_*): *f_VR_* = 1–(*f_VS_*+*f_VL_*) |  |
| *p_6_* | Proportion of immuno-compromised KA patients not responding to KA first-line treatment, derived from *p_6_* = (1-*f_T_*) *f_4_*, assuming that *f_4_* = 30% of immuno-compromised KA patients who are not killed by the treatment, do not respond to KA first-line treatment |  |
| *p_7_* | Proportion of immuno-compromised KA patients who appear to recover under KA first-line treatment but will develop PKDL later, derived from *p_7_* = (1-*f_T_*)(1-*f_4_*) *f_2_*, assuming that a fraction *f_2_* = 3% of immuno-compromised KA patients who are neither killed by the treatment nor experienced obvious treatment failure appear to recover under KA treatment but will develop PKDL |  |
| *p_8_* | Proportion of immuno-compromised KA patients recovering during first-line treatment, derived from *p_8_* = (1-*f_T_*)(1-*f_4_*)(1-*f_2_*) |  |
