# Supplementary material for: Treatment of Visceral Leishmaniasis: Model-Based Analyses on the Spread of Antimony-Resistant L. donovani in Bihar, India
Source: PLoS Negl Trop Dis. 2012 Dec 20;6(12):e1973. doi: 10.1371/journal.pntd.0001973 (PMC3527335; doi:10.1371/journal.pntd.0001973)
Supplement: Table S7 — Model parameters—resistance and fitness. (DOC) [file pntd.0001973.s009.doc]

Table S7 – Model parameters – resistance and fitness.

|  | Description |
| --- | --- |
| *r* | Scaling factor for the treatment failure rate of patients infected with resistant parasites |
| *p_1r_* | Proportion of KA patients infected with resistant parasites not responding to KA first-line treatment, derived from *p_1r_* = (1-*f_T_*)*f*_1_*_r_*, whereby *f_1r_* = *f_1_*+(1-*f_1_*) *r* of the patients who are not killed by the treatment do not respond to KA first-line treatment |
| *p_2r_* | Proportion of KA patients infected with resistant parasites who appear to recover under KA first-line treatment, but will develop PKDL later, derived from *p_2r_* = (1-*f_T_*)(1-*f_1r_*) *f_2_*, whereby *f_2_* = 3% of the patients who are neither killed by the treatment nor experienced obvious treatment failure appear to recover under KA treatment but will develop PKDL |
| *p_3r_* | Proportion of KA patients infected with resistant parasites recovering during KA first-line treatment, derived from *p_3r_* = (1-*f_T_*)(1-*f_1r_*)(1-*f_2_*) |
| *p_6r_* | Proportion of immuno-compromised KA patients infected with resistant parasites not responding to KA first-line treatment, derived from *p_6r_* = (1-*f_T_*) *f_4r_*, whereby  *f_4r_* = *f_4_*+(1-*f_4_*) *r* of the patients who are not killed by the treatment, do not respond to KA first-line treatment |
| *p_7r_* | Proportion of immuno-compromised KA patients infected with resistant parasites who appear to recover under KA first-line treatment but will develop PKDL later, derived from *p_7r_* = (1-*f_T_*)(1-*f_4r_*) *f_2_*, whereby a fraction *f_2_* = 3% of the patients who are neither killed by the treatment nor experienced obvious treatment failure appear to recover under KA treatment but will develop PKDL |
| *p_8r_* | Proportion of immuno-compromised KA patients infected with resistant parasites recovering during first-line treatment, derived from *p_8r_* = (1-*f_T_*)(1-*f_4r_*)(1-*f_2_*) |
| *f_H_* | Scaling factor for the probability that infected humans become sick |
| *f_HSr_* | Fraction of individuals infected with resistant parasites in stage *I_HDr_* who develop symptomatic KA, derived from *f_HSr_* = *f_HS_* *f_H_* |
| *f_HLr_* | Fraction of individuals infected with resistant parasites in stage *I_HDr_* who go directly to *R_HL_* and will later develop PKDL, derived from *f_HLr_* = *f_HL_* *f_H_* |
| *f_HRr_* | Fraction of individuals infected with resistant parasites in stage *I_HDr_* who recover without showing a symptomatic course of infection (*R_HD_*), derived from *f_HRr_* = 1–(*f_HSr_*+*f_HLr_*) |
| *f_VSr_* | Fraction of immuno-compromised individuals infected with resistant parasites in stage *I_VDr_* who develop symptomatic KA, derived from *f_VSr_* = *f_VS_* *f_H_* |
| *f_VLr_* | Fraction of immuno-compromised individuals infected with resistant parasites in stage *I_HDr_* who go directly to *R_VL_* and will later develop PKDL, derived from *f_VLr_* = *f_VL_* *f_H_* |
| *f_VRr_* | Fraction of immuno-compromised individuals infected with resistant parasites in stage *I_VDr_* who recover without showing a symptomatic course of infection (*R_VD_*), derived from *f_VRr_* = 1–(*f_VSr_*+*f_VLr_*) |
| *f_FS_* | Scaling factor for the probabilities *p_F3_* and *p_F4_* that a sand fly becomes infected when feeding on symptomatic hosts infected with resistant parasites |
| *f_FA_* | Scaling factor for the probabilities *p_F1_* and *p_F2_* that a sand fly becomes infected when feeding on asymptomatic hosts infected with resistant parasites |
| *f_FH_* | Scaling factor for the probabilities *p_F1_*, *p_F2_*, *p_F3_* and *p_F4_* that a sand fly becomes infected when feeding on hosts infected with resistant parasites |
| *f_HF_* | Scaling factor for the probability *p_H_* that a human becomes infected after the blood meal of a sand fly infected with resistant parasites, derived from *p_Hr_* = *f_HF_* *p_H_* |
